# COVID-19 Pandemic-Related Distress and Self-Efficacy to Manage Dementia Care Among Family Caregivers

**DOI:** 10.1093/geroni/igab046.1857

**Published:** 2021-12-17

**Authors:** Lena Thompson, Haley Schneider, Maria Donohoe, Elizabeth Saathoff, Lubna Hossain, Sato Ashida

**Affiliations:** 1 University of Iowa College of Public Health, Iowa City, Iowa, United States; 2 University of Iowa, Iowa City, Iowa, United States

## Abstract

The COVID-19 pandemic posed numerous challenges to persons with dementia (PWD) and their caregivers. To better understand these challenges, we conducted a mixed methods study analyzing data from interviews with family caregivers participating in an ongoing intervention study. Telephone interviews were conducted with 58 family caregivers of PWD diagnosed within the past two years. Participants reported self-efficacy (SE) using a 5-item scale (e.g. handle problems with memory, keep PWD at home) and rated pandemic-related distress on a 1-10 scale. They also qualitatively described effects of the pandemic on care recipients and themselves. Qualitative data were coded and organized by concepts from the Stress Process Model. Distress level ranged from 1-9 and was negatively associated with SE to manage dementia care (r=-316, p=0.036). Caregivers described distress related to primary stressors such as loss of services (respite care, assistance with daily tasks) and resistance to mask wearing by PWD due to behavioral symptoms. Secondary stressors included managing work or supervising children’s schoolwork at home while providing care. Caregivers most often expressed distress related to inability to access coping resources such as family or friends, and worried that PWD were not able to rely on their support systems. At all reported levels of distress, inability to interact with members of support networks (e.g., family, friends, service providers) was identified as most distressing. This was compounded by lower self-efficacy to manage dementia care. Efforts to decrease pandemic impacts must consider strategies to safely keep PWD and caregivers connected with family, friends, and service providers.

